# Identifying Malnutrition Risk in the Elderly: A Single- and Multi-Parameter Approach

**DOI:** 10.3390/nu16152537

**Published:** 2024-08-02

**Authors:** Karolina Kujawowicz, Iwona Mirończuk-Chodakowska, Monika Cyuńczyk, Anna Maria Witkowska

**Affiliations:** Department of Food Biotechnology, Medical University of Białystok, 15-089 Białystok, Poland; iwona.mironczuk-chodakowska@umb.edu.pl (I.M.-C.); monika.cyunczyk@umb.edu.pl (M.C.); anna.witkowska@umb.edu.pl (A.M.W.)

**Keywords:** malnutrition, elderly, CGA, MNA, sarcopenia, depression, appetite, phase angle, COVID-19, polypharmacy

## Abstract

Malnutrition is a significant concern affecting the elderly, necessitating a complex assessment. This study aims to deepen the understanding of factors associated with the assessment of malnutrition in the elderly by comparing single- and multi-parameter approaches. In this cross-sectional study, 154 individuals underwent a comprehensive geriatric assessment (CGA). Malnutrition risk was determined using the mini nutritional assessment (MNA). Additional factors assessed included sarcopenia, polypharmacy, depression, appetite, handgrip strength, and gait speed. Phase angle (PA) and body composition were measured using bioelectrical impedance analysis (BIA). The MNA identified a malnutrition risk in 36.8% of individuals. The geriatric depression scale (GDS) and PA demonstrated moderate effectiveness in assessing malnutrition risk, with AUC values of 0.69 (95% CI: 0.60–0.78) and 0.62 (95% CI: 0.54–0.72), respectively. A logistic regression model incorporating handgrip strength, skeletal muscle mass, sarcopenia, osteoporosis, depression, specific antidepressant use, mobility, appetite, and smoking achieved superior performance in predicting malnutrition risk, with an AUC of 0.84 (95% CI: 0.77–0.91). In conclusion, this study demonstrates that integrating multiple parameters into a composite model provides a more accurate and comprehensive assessment of malnutrition risk in elderly adults.

## 1. Introduction

Population aging is becoming an increasingly significant public health and social concern. The number of individuals aged 60 and above is projected to increase by 56% between 2020 and 2030, reaching 1.4 billion [1]. This demographic shift is driving the development of comprehensive geriatric care and stimulating research on the health and quality of life in this age group [2,3], but also increasing economic and social costs [4]. It is evident that both the nutritional status of the body and proper nutrition play pivotal roles in maintaining health and fitness among the elderly. Therefore, identifying the risk of malnutrition is of paramount importance [5,6].

Malnutrition among the elderly can lead to a range of adverse health outcomes, including impaired immune function, muscle loss, delayed wound healing, an increased risk of falls and fractures, cognitive decline, and an overall reduced quality of life [7,8,9,10]. Identifying and addressing the risk of malnutrition in elderly individuals is of utmost importance to promote healthy aging and prevent adverse health outcomes. Early detection of malnutrition risk allows for timely interventions, which may include nutritional counseling, dietary supplementation, and referral to appropriate healthcare professionals or community support services [11].

Malnutrition in the elderly is a complex health problem influenced by a variety of factors [12]. One such factor is a decrease in appetite [13]. It has been demonstrated that malnutrition can be a risk factor for osteoporosis [14,15]. Furthermore, depression [16,17], medication use [18,19] and lifestyle factors, such as smoking and diet [20,21], have been significantly associated with malnutrition in older people.

The prevalence of multimorbidity increases with age and consequently leads to polypharmacy [22]. Although there is an established association between polypharmacy and malnutrition [19,23], further research is needed to elucidate the relationship between malnutrition and both the number and types of medications used [24,25].

There are notable discrepancies in body composition between older and younger individuals. It is observed that physiological changes, such as an increase in adipose tissue mass and a reduction in skeletal muscle mass, occur in older individuals [26,27]. Bioelectrical impedance analysis (BIA) is a highly acceptable method for monitoring changes in body composition, assessing nutritional status, and identifying the risk of malnutrition. This method offers several advantages, including ease, speed, precision of measurement, and overall effectiveness [28,29]. 

A BIA parameter of interest is the phase angle (PA), which has been demonstrated to be a valuable tool for predicting and monitoring changes in response to therapy [30,31]. The PA reflects the quantity and quality of soft tissue [32]. A typical range for a PA value is 5–7°, which indicates good health and cellular integrity. A reduced PA may indicate structural damage to cell membranes or a reduction in cell density, signifying a limitation in cellular function [33]. 

One of the most significant issues in the context of malnutrition in older people is sarcopenia. Despite efforts to elucidate the association between sarcopenia [34,35] and/or malnutrition [35,36] and phase angle, further investigation is necessary to fully understand this relationship and its potential clinical implications for populations affected by sarcopenia and/or malnutrition. 

Malnutrition in the elderly represents a significant contemporary health challenge that necessitates a comprehensive, interdisciplinary approach [37,38,39] and careful consideration of associated factors.

The aim of this study is to gain a deeper understanding of the potential risks associated with malnutrition in the elderly population. To achieve this, we compare the effectiveness of a single-parameter approach with that of a multi-parameter approach.

## 2. Materials and Methods

### 2.1. Study Design

A cross-sectional study.

### 2.2. Data Collection

#### 2.2.1. Study Group

A random and non-probabilistic sampling method, along with selection by elimination based on age and exclusion criteria, was employed to select community-dwelling participants aged 60 and older from various settings, such as community centers, senior learning centers, and senior organizations. A total of 154 participants were ultimately included in the study. The study was designed to detect a mean effect size (d = 0.6) with a statistical power of 0.8 and an alpha level of 0.05. The power analysis indicated that a minimum of 52 participants per group (those at risk and not at risk of MNA) were required to meet the specified criteria in a two-sided *t*-test. To guarantee the reliability of our findings, the requisite sample size was adjusted, increasing by 15% to accommodate the potential utilization of non-parametric tests, and the figure was subsequently rounded up. The aforementioned meticulous calculations yielded a target sample size of 154 participants. It is our contention that this sample size exceeds the minimum requirements set out in the power analysis and is sufficient to detect the anticipated effects with an acceptable level of statistical power. A balanced number of participants in the groups of interest were recruited to ensure the reliability of the results and their applicability to the wider population.

**Figure 1:**
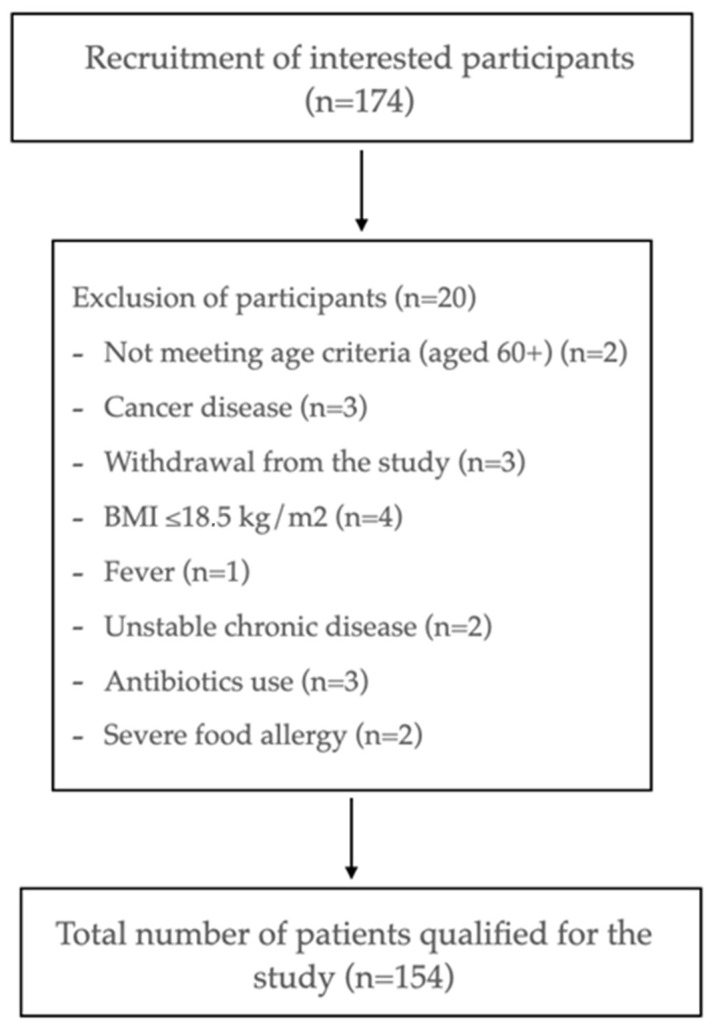
Flowchart of participant recruitment process.

The study included individuals who met the following criteria: they were either male or female, over the age of 60, and were able to walk independently without using a pacemaker. The study population comprised individuals with a stable chronic disease, defined as a long-term condition that can be managed with regular medication [40]. The exclusion criteria were as follows: a BMI of ≤18.5 kg/m^2^, sarcopenia, sarcopenic obesity, severe food allergy or intolerance to dairy products, dementia, active infectious disease (e.g., hepatitis B or C, HIV infection), recent antibiotic use, dysphagia, or severe gastrointestinal disorders (e.g., newly diagnosed coeliac disease, short bowel syndrome, pancreatic insufficiency). Additionally, individuals with uncompensated or untreated chronic diseases, end-stage liver and/or kidney failure, acute myocardial infarction within the past 30 days, or active malignant neoplasm within the past five years were excluded. The recruitment flowchart is presented in Figure 1. Polypharmacy is defined as the routine use of at least five medications per day [41]. 

The study was conducted in accordance with ethical guidelines designed to ensure that participants’ autonomy, privacy, and dignity were preserved. These measures were implemented to obtain reliable data while ensuring the safety and comfort of all involved. The study received approval from the Bioethics Committee of the Medical University of Bialystok (approval number APK.002.421.2021) and was carried out in compliance with the Declaration of Helsinki.

#### 2.2.2. Data Collection

The data were collected by a trained interviewer through the analysis of the participants’ clinical records. These records included medical history, the number of medications being taken, and the presence of any diseases.

The dataset comprised a range of variables, including age, sex, education, number of medications, height, weight, body mass index (BMI), waist–hip ratio (WHR), mid-arm circumference (MAC), right calf circumference (CC), and comorbidities (e.g., hypertension, diabetes, depression, atherosclerosis, gout, hypothyroidism, and hyperlipidemia). Additional variables included the amount and regularity of food intake, as well as leisure activities. All parameters were assessed once in the morning between 8:00 and 10:00, while participants were in a fasting state and under quiet conditions.

A comprehensive geriatric assessment (CGA) was conducted to evaluate the mental, physical, and functional health status of the study group.

#### 2.2.3. Assessment of Cognitive Performance

Cognitive performance was evaluated using the mini-mental state examination (MMSE) [42]. Scores were adjusted for age and education level according to the Mungas method: adjusted MMSE score = raw MMSE score—(0.471 × [years of education − 12]) + (0.131 × [age − 70]) [43]. The study employed the conventional cut-off point of 24 or above out of a maximum of 30 scores, as described in the literature [44,45].

#### 2.2.4. Evaluation of Depressive Conditions

The mental state of the patients was assessed using the geriatric depression scale (GDS) [46], a validated tool designed to evaluate depressive conditions in older adults. The study utilized the original 30-item version, which is widely recommended for screening depression in this population [47,48,49]. The clinical utility of this version was considered adequate for screening purposes [49]. In the present study, a score of 0–9 was considered indicative of normal mental health, while a score of 10–19 suggested the presence of mild depression [46].

#### 2.2.5. Vital Signs Assessment

The following measurements were taken: temperature measured using a contact-free thermometer (HeTaiDa HTD8808C); blood pressure measured using a medical blood pressure monitor (Omron M6 Comfort HEM-7360-E); and SpO2 saturation measured using a standard pulse oximeter with a finger probe (Medical Pulse Oximeter Contec Cms50d). Any abnormal results (e.g., fever, low SpO2) were duly recorded, and participants with fever were excluded from the study.

#### 2.2.6. Assessment of the Ability to Perform Daily Living Activities

The Barthel scale was used to assess functional independence due to its well-validated status and perceived superiority among commonly used indexes of activities of daily living (ADL). This scale was specifically employed in the study to evaluate participants’ ability to perform daily living activities [50]. 

The individual’s capacity to perform instrumental activities of daily living (IADL) within the domestic environment was evaluated using Lawton’s instrumental activities of daily living scale (IADL) [51]. This assessment tool determines the participant’s ability to function independently in daily life. The IADL scale comprises nine questions covering a range of daily activities, including medication administration, telephone use, and household tasks, such as shopping, cooking, cleaning, and laundry [52]. Additionally, the scale is validated for assessing dementia in older individuals [53]. 

#### 2.2.7. Assessment of Physical Fitness

The degree of mobility was assessed using the timed up-and-go (TUG) test. This test involved measuring the fluidity of gait over a distance of 3 m at individual stages: standing up from a chair, walking 3 m, turning 180 degrees, returning, and sitting down again [54]. Participants were requested to complete the gait test on two occasions.

A grip strength (GS) test was employed to evaluate physical functionality. Dominant grip strength (in kilograms) was measured using a hand-held dynamometer (JAMAR^®^ PLUS + Hand Dynamometer) in a seated position, following clinical practice recommendations for older individuals [55]. Measurements were taken twice for each arm, and the values were averaged to obtain a single mean value. Arm circumference (in centimeters) was measured at the thickest part of the arm using a tape measure. 

In accordance with the most recent recommendations and definition of sarcopenia by the European Working Group on Sarcopenia in Older People (EWGSOP2), the strength, assistance in walking, rise from a chair, climb stairs, and falls (SARC-F) questionnaire was employed to identify individuals at risk of sarcopenia. The SARC-F test used validated cut-offs: ≥4 for those at risk of sarcopenia and <4 for those without sarcopenia risk [5,56,57,58]. Whole-body skeletal muscle mass (SMM) and appendicular skeletal muscle mass index (ASMI) were calculated using bioelectrical impedance analysis (BIA) according to the ASM/height^2^ formula. The incidence of low ASMI was determined using the EWGSOP2 cut-off values: ≤7.0 kg/m^2^ for males and ≤5.5 kg/m^2^ for females [59]. Muscle strength was assessed through handgrip strength measurement with cut-off points of <27 kg for men and <16 kg for women. Physical performance was evaluated using the timed up-and-go (TUG) test, with a cut-off value of ≥20 s. In accordance with EWGSOP2 recommendations, low gait speed was excluded as a diagnostic criterion for sarcopenia, as its inclusion resulted in a lower incidence of sarcopenia [5]. During the study, no individual exhibited all the criteria necessary for a diagnosis of sarcopenia.

Sarcopenic obesity (SO) was defined as the co-occurrence of sarcopenia and obesity. The diagnosis of sarcopenia was made according to the criteria set forth by the European Working Group on Sarcopenia in Older People (EWGSOP2). Specifically, the appendicular skeletal muscle mass (ASM) was less than 5.5 kg/m^2^ for women and less than 7.00 kg/m^2^ for men, while the handgrip strength (HGS) was less than 16 kg for women and less than 17 kg for men [5,60]. Obesity was determined by a body mass index (BMI) exceeding 30 kg/m^2^ [61,62,63,64,65,66,67]. None of the study participants exhibited evidence of sarcopenic obesity.

The risk mortality and disease index was calculated based on a study by Siervo et al. [68,69]. This study demonstrated that the relationship between visceral fat and ASMI (FM/ASMI) is a more effective predictor of mortality and diabetes risk compared to the simpler FM/FFM index. 

#### 2.2.8. Assessment of Nutritional Status

Nutritional status was evaluated using the mini nutritional assessment—long form (MNA-LF), which involved direct questioning of patients [70]. The assessment comprised 18 items, including anthropometric measurements and questions pertaining to dietary intake, appetite, general health, and disability status. The possible scores range was from 0 to 30. A score below 17 indicates malnourishment, a score of 17–23.5 indicates a risk of malnutrition, and a score of 24 or higher indicates satisfactory nutritional status [71,72]. In the present study, individuals with an MNA score between 17 and 23.5 were classified as being at risk of malnutrition, and a score of 24 or higher indicates satisfactory nutritional status.

#### 2.2.9. Appetite Assessment

Appetite was evaluated using the simplified nutritional appetite questionnaire (SNAQ) and the Council on Nutrition Appetite questionnaire (CNAQ) [73]. The SNAQ comprises four items assessing appetite, feelings of satiety, taste of food, and the number of meals consumed per day. It is designed as a self-assessment tool that is straightforward and rapid to administer, requiring no specialized training or laboratory tests. The rating scale ranges from 4 to 20 points. Previous validation studies indicate that a score of 14 or below may suggest malnutrition and involutional weight loss [74,75,76]. Additionally, the CNAQ was employed for appetite assessment. Studies have shown that both the CNAQ and SNAQ are highly accurate and effective in predicting malnutrition in older populations across various levels of specialization [71,77,78].

#### 2.2.10. Anthropometric Measurements

Body weight was measured using a stationary SECA scale. Body mass index (BMI) was calculated from the body weight (kg) and height (cm). These measurements were taken by qualified personnel. In the assessment of obesity, the World Health Organization (WHO) primarily uses BMI as a criterion. Specifically, a BMI of at least 30 kg/m^2^ is considered indicative of obesity across all age and gender groups in Caucasians.

According to WHO recommendations, the following age- and gender-specific BMI cut-off points were applied:

Normal weight: Up to 60 years old with a BMI ≤ 24.9 kg/m^2^;

Overweight: Above 60 years old with a BMI ≥ 25.0 kg/m^2^;

Obesity: Above 60 years old with a BMI ≥ 30 kg/m^2^.

For individuals aged 65 years or older, the classification criteria were as follows:

Normal weight: BMI of 24.0–29.9 kg/m^2^;

Overweight: BMI ≥ 30 kg/m^2^;

Obesity: BMI ≥ 35 kg/m^2^ [79].

The waist–hip ratio (WHR) was calculated by dividing the waist circumference at the narrowest point above the navel by the hip circumference at the widest point. To determine the appropriate criteria, the two WHO reports on abdominal obesity were consulted. According to these reports, a waist circumference of 88 cm or above in women and 102 cm or above in men indicates a markedly elevated risk of developing metabolic complications [79,80].

In this study, silhouette types were classified according to the World Health Organization (WHO)’s recommendations for waist circumference and waist-to-hip ratio (WHR). An android body shape, often associated with an “apple” figure, was defined by a WHR of 0.8 or greater in women and 1.0 or greater in men. Conversely, a gynoid silhouette, commonly referred to as a “pear” type, was indicated by a WHR of 0.8 or less in women and 1.0 or less in men. Silhouettes that closely matched the ideal proportions had a WHR of 0.7 in women and 1.0 in men [81].

#### 2.2.11. Body Composition

Body composition was quantified using the bioelectrical impedance analysis (BIA) method. The SECA mBCA 525, a validated medical body composition analyzer with an 8-point BIA system, was employed for measurements. This device utilizes proprietary predictive formulas derived from research to calculate parameters such as total body water (TBW), extracellular water (ECW), fat-free mass (FFM), relative fat mass (RFM), and skeletal muscle mass (SMM) across various body areas, including arms, legs, torso, and the whole body. Additionally, total body fat (TBF) and visceral adipose tissue (VAT) were assessed. Energy was quantified through indirect calorimetry, facilitated by a body composition analyzer. Energy was expressed in three parameters: resting energy expenditure (REE), the amount of energy required by a body to maintain its respiratory, digestive, cardiovascular, and other vital systems in rest; total energy expenditure (TEE), the total amount of energy required by an individual on a daily basis, encompassing both physical activity and REE; and total energy content (TEC), calculated from the fat, protein, and carbohydrate content in the body using the following equation: Total Energy Content (TEC) = (Fat Mass × 9 kcal) + (Protein Mass × 4 kcal) + (Carbohydrate Mass × 4 kcal). Analyses using the SECA mBCA 525 identified standards for several parameters, including bioelectrical impedance vector analysis (BIVA), fat mass indices (FMI, FMMI), phase angle (φ), fat mass (FM), and skeletal muscle mass (SMM) [82,83]. The phase angle was calculated directly from resistance (R) and reactance (Xc) using the formula [32]:

Phase angle = arctan (Xc-/-R) × (180/π).

To achieve optimal normalization in the BIA test, it is recommended to perform the test after the participant has been supine for 10 min [84]. This is because bioelectrical impedance increases when individuals lie on their backs, due to the displacement of body fluids from the extremities to the thorax [85]. Parameters were measured with the participant in the supine position using eight self-adhesive electrodes placed on specific body parts. The device measured impedance separately for the right arm, left arm, torso, right leg and left leg, right half of the body, and left half of the participant’s body at nine different frequencies (1, 2, 5, 10, 20, 50, 200, and 500 kHz). The SECA mBCA 525 utilized a measurement current of 100 μA with a maximum measurement time of 30 s. It is the only instrument validated against magnetic resonance imaging (MRI), the 4C method (four-compartment model), and whole-body dilution techniques using sodium bromide (NaBr) and deuterium oxide (D_2_O) [82]. Additionally, the device has been validated for use across different ethnic groups and for individuals with obesity (BMI > 30 kg/m^2^) [86]. The validation process adhered to rigorous methodological standards, confirming the device’s accuracy and reliability. It provides a comprehensive repository of multi-ethnic data from over 3000 individuals across Germany, Japan, and Mexico. This extensive dataset supports a thorough assessment of body composition in diverse populations [83,87]. A validation study demonstrated that the data from the SECA mBCA 525 are applicable for clinical purposes, including identifying individuals at increased risk of adverse events and monitoring their response to treatment [88]. The fat-free mass index (FFMI) was calculated using the formula FFM/(height)^2^, in accordance with the European Society for Clinical Nutrition and Metabolism (ESPEN) guidelines [89].

#### 2.2.12. Statistical Analysis

For numeric variables deviating from normality, data were reported using the median (Mdn) and the first (Q1) and third (Q3) quartiles. Categorical variables were presented as counts (n) and corresponding percentages (%).

Differences between two independent groups concerning numeric variables were assessed using the Wilcoxon rank-sum test. For categorical variables, Pearson’s chi-square test or Fisher’s exact test were applied, depending on the expected counts in each category.

The optimal cutoff values for numerical parameters were determined by maximizing the sum of sensitivity and specificity. To evaluate the discriminatory capacity, various classification metrics were calculated, including accuracy, specificity, sensitivity, and the Area Under the Curve (AUC). 

The impact of multiple parameters on the binary outcome variable (risk of MNA) was investigated using a generalized regression model with a logit link function. Variables included in the final model were selected based on the Akaike Information Criterion (AIC) using a stepwise algorithm with backward elimination.

To assess model generalizability, a 10-fold cross-validation procedure was conducted. This method determined the mean accuracy and Kappa statistics, providing insights into the models’ reliability and consistency across different data samples.

Collinearity among explanatory variables was assessed using the Variance Inflation Factor (VIF). VIF values below 3.0 were considered indicative of low collinearity, suggesting that these variables do not introduce substantial bias into the results.

The model fit was evaluated using three types of tests: the Hosmer–Lemeshow test, z-score-based tests, and χ^2^-based tests. For a comprehensive list of these tests, please refer to Table A3 in the Appendices section.

The efficacy of the statistical model was evaluated through an analysis of the receiver operating characteristic (ROC) curve and the area under the curve (AUC). Statistical significance of differences in AUC values was determined using the DeLong test and a bootstrapping procedure with 2000 replicates. The significance level was set at α = 0.05. All analyses were conducted using R statistical software (version 4.3.1; R Core Team, 2023).

## 3. Results

A total of 154 elderly individuals, aged between 60 and 80 years, were analyzed. Of these, 58 individuals (36.8%) were identified as being at risk of malnutrition according to the mini nutritional assessment (MNA), while 96 individuals (63.2%) were not at risk. The demographic characteristics of the participants according to the risk of malnutrition, categorized by their risk of malnutrition as determined by the MNA tool, are presented in Table 1. 

A comparison between the malnutrition risk groups (at risk vs. not at risk) revealed no statistically significant differences in gender, age, marital status, level of education, smoking habits, or sleep duration. However, significant differences were observed in the prevalence of certain comorbidities and medication use. Specifically, the at-risk group showed higher rates of depression (15.52% vs. 5.21%, *p* = 0.031) and osteoporosis (17.24% vs. 6.25%, *p* = 0.030) compared to the non-at-risk group. 

Additionally, the use of selective serotonin reuptake inhibitors (SSRIs) and monoamine oxidase inhibitors (MAOIs) was significantly associated with the risk of malnutrition. Individuals at risk of malnutrition were more likely to use these medications than those not at risk (18.97% vs. 6.25%, *p* = 0.015). No significant differences were observed regarding the efficacy of the other medications and diseases.

### 3.1. Physical Fitness and Body Composition

A detailed analysis of various aspects of physical fitness and body composition is presented in Table 2.

In the evaluation of malnutrition risk across different groups, several physiological and anthropometric measures showed minimal to no significant differences. Measures such as grip strength, prevalence of abdominal obesity (approximately 46.75% across groups), body mass index (BMI), waist-to-hip ratio (WHR), mid-arm circumference (MAC), and right calf circumference did not reveal significant differences between the at-risk and non-risk groups. Additionally, no significant differences were observed in muscle mass levels, fat distribution types, or visceral adipose tissue between the groups. 

Significant differences were observed in body composition indices between the groups. The at-risk group demonstrated notably lower levels of both fat-free mass (41.01 kg vs. 44.72 kg, *p* = 0.037) and skeletal muscle mass (18.35 kg vs. 20.98 kg, *p* = 0.014) compared to the non-risk group. Additionally, the mean appendicular skeletal muscle mass index (ASMI) was lower in the at-risk group (7.27 vs. 7.95, *p* = 0.020).

Individuals at risk of malnutrition also exhibited significantly lower total body water compared to those without malnutrition risk (31.11 L vs. 34.00 L, *p* = 0.041). Furthermore, the phase angle was significantly higher in the non-risk group than in the at-risk group (5.49° vs. 5.15°, *p* = 0.008). 

### 3.2. Energy, Metabolism, and Nutritional Assessment

Table 3 presents data on energy and metabolism parameters, as well as nutritional and functional assessments, for a cohort of elderly patients.

No statistically significant differences were observed between the groups concerning the energy parameters, including TEE, REE, and TEC.

Significant differences were observed in the geriatric depression scale (GDS) scores, with individuals at risk of malnutrition displaying markedly higher scores compared to those not at risk (7.50 vs. 3.0, *p* = 0.001).

Additionally, functional mobility, as measured by the timed up-and-go (TUG) test, was significantly slower in the malnutrition risk group (10.59 s vs. 9.51 s, *p* = 0.015).

Significant differences were observed in the prevalence of malnutrition risk factors, as indicated by reduced SNAQ and CNAQ scores. Individuals at risk of malnutrition had significantly lower scores on both the SNAQ (44.83% vs. 20.83%, *p* = 0.002) and CNAQ (65.52% vs. 30.21%, *p* = 0.001), compared to those not at risk.

Sarcopenia, as assessed by the SARC-F test, was also notably more prevalent among those at risk of malnutrition compared to those not at risk (18.97% vs. 4.17%, *p* = 0.003).

### 3.3. Determination of Optimal Cut-Off Points for Continuous Parameters Influencing Malnutrition Risk

In this section, we present the optimal cut-off points for various continuous parameters that influence malnutrition risk, including GS, WHR, BMI, MAC, CC, RFM, FFM, SMM, PMMTW, ASMI, TBW, ECW, FFMI, FMI, PA, VAT, TEE, REE, GDS, and TUG. Table 4 presents these cut-off points along with the corresponding metrics, which aid in evaluating the effectiveness of each parameter in distinguishing between individuals at risk of malnutrition and those who are not. 

#### The Overall Characteristic of Classification Metrics Results

The classification indices for the parameters presented in Table 4 exhibit considerable variability and demonstrate disparate performance in terms of accuracy, sensitivity, specificity, and AUC.

The observed accuracy ranged from 0.49 to 0.67. Notably, the GDS score exhibited a relatively high accuracy of 0.67, while the ASMI achieved a similarly high value of 0.66. The remaining parameters followed a similar pattern, oscillating around a threshold of 0.50, which is indicative of low accuracy.

The sensitivity values exhibited a range from 0.22 to 0.86. The PA demonstrated high sensitivity values and a strong ability to correctly identify individuals at risk of malnutrition (0.86).

The specificity values ranged from 0.31 to 0.85. Parameters such as right CC (0.85), MAC (0.84), ASMI (0.81), and FFMI (0.81) demonstrated high specificity, indicating their effectiveness in distinguishing between individuals with and without malnutrition risk. The remaining parameters exhibited lower specificity.

The AUC values for the included parameters ranged from 0.47 to 0.69. The highest AUC value (0.69) was demonstrated by the GDS score, indicating relatively good discriminatory power. Additionally, parameters such as FFM (0.60), SMM (0.62), ASMI (0.61), and total water content (0.60) exhibited average discriminatory power. The remaining parameters displayed AUC values approximately between 0.50 and 0.60, indicating limited utility in distinguishing between individuals at risk of malnutrition.

### 3.4. Identification of Parameter Profiles Influencing the Risk of Malnutrition in a Multivariate Approach

A backward stepwise algorithm was applied to 44 competitive parameters, excluding several due to multicollinearity during the preprocessing stage (refer to Table A1 in the Appendix for full details). The application of the stepwise algorithm reduced the number of parameters to nine, as detailed in Table 4, resulting in a decrease in the Akaike Information Criterion (AIC) from an initial value of 219.47 to a final value of 166.31.

The selection of the 44 parameters for analysis was derived from an inclusive approach, initially incorporating all variables in the regression model. Subsequently, a stepwise algorithm was employed to progressively select the most relevant parameters. This method proved to be more beneficial compared to the approach of selecting only statistically significant parameters.

All predictors in the final model have a variance inflation factor (VIF) coefficient below 3.0 (refer to Table A2 in the Appendix), indicating a low level of multicollinearity among the predictors. The non-significant results from the validation tests listed in Table A3 demonstrate that the model fits the observed data well. Furthermore, the cross-validation results indicate that the model has a reasonably good fit and performs well in classifying instances, achieving an average accuracy of 76.13% and moderate agreement beyond chance, as indicated by a Kappa statistic of approximately 0.472. Collectively, these results suggest that the model is well-suited for practical application and can be trusted to provide consistent and accurate predictions.

The results of the regression model, as presented in Table 5, indicate that approximately 34.4% of the variance in malnutrition risk can be attributed to the included predictors. This represents a moderate effect size within the context of clinical research.

The baseline probability of malnutrition, as assessed by the MNA scale, for patients who do not smoke, do not have osteoporosis, do not take SSRIs/MAOIs, have an average grip strength of 20.15 kg, a skeletal muscle mass of 19.33 kg, a GDS score of 5.0, a timed up-and-go (TUG) test result of 9.61 s, and exhibit no risk of malnutrition as well as lower appetite (CNAQ) or risk of sarcopenia (SARC-F), was 0.14, corresponding to a probability of 12.3%. This finding was highly significant.

The analysis revealed that smoking status is a significant predictor of malnutrition risk. Specifically, individuals who smoke exhibited significantly higher odds of being at risk of malnutrition compared to non-smokers, with an odds ratio (OR) of 7.08 (95% confidence interval: 1.90–28.65, *p* = 0.004). This underscores the substantial role that smoking plays as a major risk factor for malnutrition in elderly individuals. 

The presence of osteoporosis has been identified as another significant predictor of malnutrition risk. Individuals with osteoporosis exhibit nearly five times the odds of being at risk of malnutrition compared to those without this condition (OR = 4.89, 95% CI: 1.34–19.49, *p* = 0.019). This strong association suggests that osteoporosis may be linked to underlying nutritional deficiencies that contribute to both malnutrition and deteriorating bone health.

The intake of selective serotonin reuptake inhibitors (SSRIs) and monoamine oxidase inhibitors (MAOIs), commonly prescribed for depression and other mood disorders, is significantly associated with an increased risk of malnutrition (OR = 4.42, 95% CI: 1.11–19.64, *p* = 0.040). This finding suggests that these medications may contribute to nutritional challenges in the elderly.

Conversely, higher skeletal muscle mass is significantly associated with a reduced risk of malnutrition (OR = 0.86, 95% CI: 0.76–0.95, *p* = 0.008). This highlights the protective role of maintaining adequate muscle mass in mitigating malnutrition risk.

The results indicate that the geriatric depression scale (GDS) score and the timed up-and-go (TUG) test result are marginally associated with an increased risk of malnutrition. Both variables approach the threshold of statistical significance, suggesting that greater depressive symptoms and reduced physical mobility may contribute to a higher risk of malnutrition, though the association is not as strong as other predictors.

Categorical predictors also play a significant role in determining malnutrition risk. Individuals identified as being at risk of malnutrition by the mini nutritional assessment (MNA) and who reported lower appetite as measured by the Council on Nutrition Appetite Questionnaire (CNAQ) have significantly higher odds of malnutrition (OR = 4.34, 95% CI: 1.89–10.46, *p* = 0.001). Additionally, those classified as having sarcopenia according to the SARC-F questionnaire also demonstrate markedly increased odds of malnutrition (OR = 5.95, 95% CI: 1.29–31.47, *p* = 0.026).

These findings underscore the importance of assessing appetite and sarcopenia risk, alongside other predictors, to better identify and address malnutrition risk among elderly individuals.

#### Comparative Analysis of ROC Curves for Competing Models

**Figure 2:**
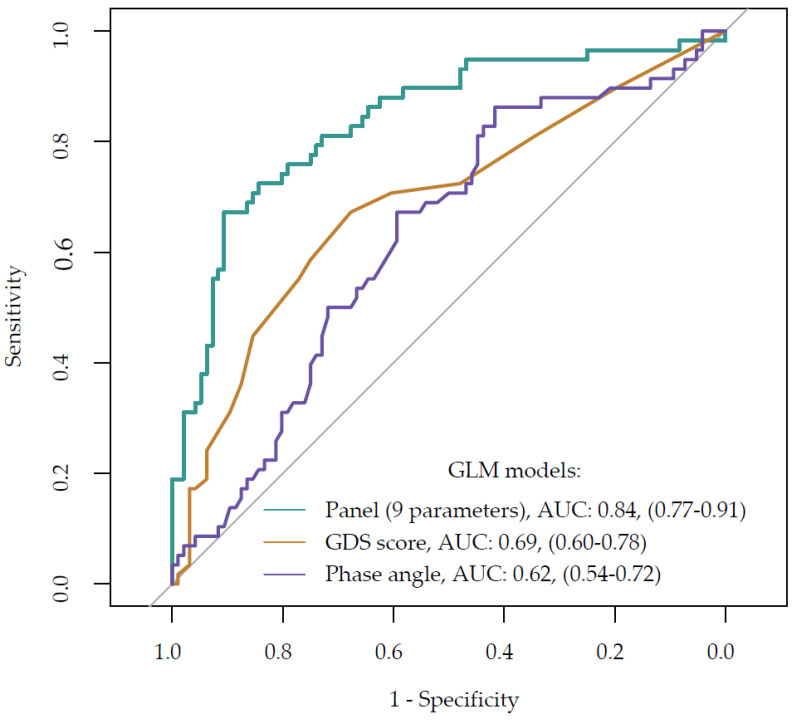
Comparative analysis of ROC curves and AUC results for multivariate regression model parameters versus key univariate parameters from optimal cutoff analysis.

Figure 2 illustrates the performance of the fitted model through its area under the curve (AUC). For comparison, the ROC curves of two individual parameters with the highest AUCs are also presented: the geriatric depression scale (GDS) score, noted for its well-balanced metrics; and the phase angle (PA), which demonstrates the highest specificity.

The DeLong test results revealed that the AUC for the composite panel of parameters was significantly higher (AUC = 0.69, 95% CI: 0.60–0.78) compared to the individual GDS score (z = 3.39, *p* = 0.001) and PA (z = 4.32, *p* < 0.001). These findings were consistent with the bootstrap test results. In contrast, no significant difference in ROC performance was found between the GDS score and phase angle (z = 0.96, *p* = 0.338).

Univariate parameters, including the geriatric depression scale (GDS) score (cut-off ≥ 5.00, AUC = 0.69, 95% CI: 0.60–0.78) and phase angle (cut-off ≤ 5.72, AUC = 0.62, 95% CI: 0.54–0.72), demonstrated superior performance in assessing the risk of malnutrition as determined by the mini nutritional assessment (MNA) in the elderly, compared to random guessing. However, the assessment’s accuracy was significantly enhanced when employing a panel of nine parameters, which achieved an AUC of 0.84 (95% CI: 0.77–0.91).

Note: Figure 2 illustrates the predictive efficacy of three models in identifying individuals at risk of malnutrition. The phase angle (PA), represented as a single predictor, demonstrated moderate discriminatory power with an AUC of 0.62. The geriatric depression scale (GDS) score, another single predictor, showed higher efficacy with an AUC of 0.69. However, the nine-parameter panel model exhibited the highest discriminatory power, achieving an AUC of 0.84. The ROC curves presented in Figure 2 indicate that the nine-parameter model is the most effective predictor of malnutrition risk among the models assessed in this study.

The findings indicate that several parameters should be considered when assessing malnutrition risk in the elderly, including smoking status, the presence of osteoporosis, and the intake of selective serotonin reuptake inhibitors (SSRIs) or monoamine oxidase inhibitors (MAOIs). Additionally, factors such as average grip strength (measured in kilograms from both the right and left hands), skeletal muscle mass (in kilograms), the geriatric depression scale (GDS) score, results from the timed up-and-go (TUG) test, the Council on Nutrition Appetite questionnaire (CNAQ) score, and the SARC-F score should also be taken into account.

## 4. Discussion

The objective of this study was to assess the risk of malnutrition and its predictors among older individuals residing in the Polish community. To achieve this, the study utilized the mini nutritional assessment (MNA) tool, which is validated for assessing malnutrition risk in the elderly and has demonstrated high sensitivity (≥80%) and good specificity (≥60%) [90]. This tool is particularly useful in various clinical settings, including comprehensive geriatric assessments (CGA) [91,92]. In this study, the MNA tool was employed to identify patients who were at risk of malnutrition. The MNA was employed to identify patients at risk of malnutrition, resulting in 36.8% of participants being classified as at risk. This prevalence is consistent with findings reported by other studies [93,94]. 

The current study found no significant association between sex or age and the risk of malnutrition, consistent with findings from other research [95,96]. This suggests that the incidence of malnutrition may not be solely influenced by gender differences but rather by individual characteristics such as educational background, socioeconomic status, health conditions, and lifestyle factors [97]. Although no differences in educational attainment or socioeconomic status were observed in this study, the presence of diseases affecting mental and physical health status, the use of specific medications, and various lifestyle factors were found to be associated with an increased risk of malnutrition among older individuals.

Current epidemiological evidence suggests that assessing the prevalence of malnutrition or its risk requires consideration of multiple factors, including location, social environment, and concomitant diseases [98,99]. Additionally, the methods and screening tools used in these assessments are crucial [100,101]. There is growing interest among researchers in developing and standardizing new methods and instruments to identify individuals at risk of malnutrition and in integrating these with existing tools and data [102]. 

In recent years, instrumental methods such as phase angle measurement have gained prominence in assessing malnutrition risk among older individuals [30,103]. A study investigating phase angle levels in this population suggested that lower phase angle values in women might be linked to reduced muscle mass compared to men. However, the study ultimately found no significant gender differences in phase angle [85]. While reference ranges for phase angle can vary based on population characteristics, age, and gender, our study indicates that phase angle may serve as a useful predictor of malnutrition risk across the general population, regardless of gender.

In recent years, the interplay between malnutrition, sarcopenia, cachexia, and phase angle has been extensively studied [104]. Bellido et al. proposed a hypothesis that imbalances between body cell mass (BCM) and tissue hydration might be linked to specific mechanisms affecting these conditions [30]. Their research identified that malnutrition and sarcopenia lead to reductions in BCM and cell membrane surface area, which can decrease reactance and BCM. This reduction manifests as a shift of the bioelectrical impedance vector along the X-axis, resulting in a decrease in phase angle (PA). Additionally, individuals at risk of malnutrition were found to have significantly lower total body water content compared to those without malnutrition risk. This finding suggests that PA could serve as an indicator of fluid balance, reflecting alterations in cell membrane integrity, and might also indicate the severity and prognosis of heart failure [105].

The present study demonstrated that phase angle, as a univariate parameter, exhibited superior performance in assessing the risk of malnutrition, as determined by the MNA scale, in elderly individuals without concurrent sarcopenia. Phase angle was more effective than a randomized alternative parameter. A significant difference in phase angle was observed between those at risk of malnutrition (5.15°) and those not at risk (5.49°). The optimal cut-off point for predicting malnutrition risk across the entire population was identified as 5.72°. Additionally, the study found a significant correlation between phase angle and malnutrition in both male and female participants, with lower cut-off points of 4.03° for males and 3.65° for females [106]. The phase angle used in the assessment of malnutrition, was found to be a slightly higher in males compared to females [107,108]. These findings may align with studies suggesting that phase angle (PA) increases proportionally with body mass index (BMI), particularly in individuals with higher muscle and fat cell counts when BMI is below 30 kg/m^2^. Consequently, lower PA values could indicate a lower muscle cell count and a higher proportion of fat cells [88]. Individuals with a BMI of 30 kg/m^2^ or above often experience an expansion of extracellular fluid. This increase in fluid volume raises electrical resistance and, consequently, results in a lower phase angle [109]. Additionally, at higher BMI values, the increased production of adipokines by adipose tissue can lead to cellular damage and reduced cellular reactivity, which further contributes to a decrease in PA [110].

Consequently, PA serves as a composite measure reflecting both body fat and muscle mass [111]. Research indicates that older women typically have a higher BMI and a greater percentage of body fat, along with lower muscle mass, compared to men [112]. This may result in reduced physical activity levels among women [113]. Wirth et al. reported a lower phase angle in women compared to men (4.1 ± 1.1° vs. 4.4 ± 1.2°), although this gender difference was eliminated after adjusting for age [114]. These studies suggest that gender-specific data in PA assessments for the elderly may be contentious due to natural variations in body composition with age. Based on the findings presented, it is advisable to establish a uniform measurement standard for PA, regardless of gender, to improve the comparability of results across older individuals. Additionally, considering that age and health status significantly influence body composition in older adults, a standardized approach to PA measurement could enhance the accuracy of longitudinal studies and provide a clearer understanding of its impact on health and functional capacity.

The present study also revealed that patients at risk of malnutrition were significantly more likely to have osteoporosis. This finding extends previous research that identified phase angle as a predictor of osteoporosis, independent of age and gender. Prior studies have shown that a lower phase angle is associated with an increased risk of osteoporosis [115]. 

A substantial body of research has highlighted the association between the risk of malnutrition and/or undernutrition and sarcopenia, or the risk of sarcopenia, in community-dwelling older adults [116,117,118,119]. Estimates suggest that the prevalence of sarcopenia is around 19% [120]. Although this study did not determine sarcopenia prevalence according to EWGSOP2 criteria, it did reveal a significant difference in the appendicular skeletal muscle mass index (ASMI) between those at risk of malnutrition (7.27 kg/m^2^) and those not at risk (7.95 kg/m^2^). Additionally, the risk of sarcopenia, as assessed by the SARC-F questionnaire, was significantly higher among those at risk of malnutrition (18.97%, *p* = 0.003). It is crucial to note that the coexistence of malnutrition and sarcopenia may increase mortality risk more than either condition alone, underscoring the clinical importance of accurately diagnosing and monitoring both disorders [121].

Although obesity in older adults is associated with increased risk of cardiovascular disease [122], stroke [123], cancer [124], and dementia [125,126], which negatively impact quality of life and functioning, some research suggests that older individuals with obesity may experience lower cardiovascular mortality compared to their younger counterparts [127]. The ‘obesity paradox’ in older age remains a subject of debate and is often evaluated using BMI. However, other factors, such as lean body mass, fat distribution, and cardiac fitness, might also play crucial roles in survival among obese older individuals [128,129]. Mezian et al. suggest that while obesity may be linked to a lower risk of certain metabolic diseases, it may also contribute to muscle loss and sarcopenia in the elderly [130]. Our findings indicate that, despite the prevalence of obesity among those at risk for malnutrition, these individuals show a significantly higher risk of sarcopenia, along with notably lower skeletal muscle mass, lean mass, and ASMI. 

The aging process is frequently accompanied by multiple comorbidities [131], which often necessitate the use of several medications for management [132,133]. While a precise definition of polypharmacy varies, the World Health Organization (WHO) defines it as the regular use of five or more medications, including prescription drugs, over-the-counter medications, herbal remedies, and dietary supplements [41]. This practice can be linked to increased hospital admissions and higher costs associated with patient care and health system operations [134]. Data suggest that patients who use an average of five medications commonly experience at least one serious medication-related problem [135,136]. Polypharmacy remains a significant health challenge for older individuals [137]. Some researchers suggest that multimorbidity in older adults often leads to polypharmacy [138]. A cohort study of hospitalized older patients found that those with malnutrition had a higher Charlson Comorbidity Index score compared to those without malnutrition [139]. In our study, polypharmacy was more prevalent among those at risk for malnutrition, with 41% of this group affected compared to 32.4% of the overall study population. However, this difference was not statistically significant. It is important to note that as the number of medications increases, so does the risk of malnutrition, which may subsequently elevate the risk of mortality [18,140].

To date, studies have identified an association between malnutrition in the elderly and the use of certain medications, including anti-cancer medications [141], hypotensive drugs, proton pump inhibitors, and anti-anxiety medications [142]. Selective serotonin reuptake inhibitors (SSRIs), commonly prescribed for depression in older individuals [143], impact the 5-hydroxytryptamine 2C (5HTR2C) receptor in the hypothalamus, which regulates appetite [144]. While SSRIs may reduce impulsivity and food intake in the short term by increasing feelings of satiety through enhanced metabolic and sympathetic activity, long-term use (≥12 months) can lead to weight gain due to increased cravings for carbohydrates [145,146]. Monoamine oxidase inhibitors (MAOIs), used for treatment-resistant major depressive disorder (MDD) when SSRIs are ineffective, may also affect body weight and nutritional status [147]. This study is the first to highlight an association between malnutrition risk and the use of SSRIs and MAOIs. It is essential to recognize that these medications can have varying effects on body weight depending on the duration of use, potentially leading to adverse changes in patients’ weight and nutritional status [148]. 

The association between malnutrition risk and depression is well documented in the literature. Nevertheless, the direction of causality between these two conditions remains uncertain. It is unclear whether depression precedes malnutrition or if malnutrition results from depressive symptoms [149]. Available evidence suggests that depression may increase the risk of malnutrition in older individuals, highlighting the complex interplay between these conditions [150]. Kirk et al. demonstrated that depressive symptoms are independently associated with sarcopenia and malnutrition in older individuals [151], underscoring the importance of screening older adults at risk of sarcopenia and malnutrition for depression at an early stage [152]. The findings of this study support the hypothesis that there is an association between malnutrition risk and depressive symptoms in older individuals, as evidenced by both the GDS score and the observed prevalence of depression. It is important to note that the study participants did not exhibit cognitive impairment, which could otherwise be a significant factor influencing depressive symptoms [153]. 

A cross-sectional study found that 27.5% of patients aged over 65 diagnosed with COVID-19 were at risk of malnutrition, while 52.7% were classified as malnourished [79]. In contrast, our study observed that 39.66% of patients at risk of malnutrition had a history of COVID-19. However, our results revealed no statistically significant association between a past COVID-19 infection and malnutrition risk. This suggests that a previous COVID-19 infection may not directly influence the risk of malnutrition.

It has been proposed that smoking may act as a confounding factor and contribute to weight loss, potentially leading to malnutrition in older individuals [154]. Additionally, other studies have identified several moderate determinants of malnutrition, including poor appetite [20], hospitalization [155,156], poor self-rated health [157,158,159], and low physical fitness [20,160]. 

It is also possible that cognitive function [154,161], depression, or depressive symptoms, as assessed by the GDS [154,162], may influence malnutrition; however, further research is needed to explore these potential relationships.

The results of our study indicated several factors, including smoking, depressive symptoms, appetite disorders, and the intake of SSRI/MAOI medications, were significant predictors of malnutrition risk. The model incorporating smoking, osteoporosis, SSRI/MAOI intake, handgrip strength, GDS, TUG, skeletal muscle mass (SMM), CNAQ, and SARC-F achieved a high accuracy with an AUC of 0.84 (95% CI: 0.77–0.91) in predicting malnutrition risk. Smoliner et al. [163] also identified depression, as assessed by the GDS, as the sole independent risk factor for malnutrition, with age, gender, level of care, number of prescriptions, and the ability to self-care showing no significant impact. Nevertheless, the researchers recommend caution in interpreting these data, as the relationship between depression and malnutrition remains unclear.

A number of studies have corroborated the correlation between malnutrition and various aspects of physical functioning, including handgrip strength [164,165,166], walking speed as assessed using the TUG test [167,168,169], appetite using the CNAQ [170,171,172], and skeletal muscle mass [96,167]. Nevertheless, there are studies that challenge this for handgrip strength [173,174]. Conversely, SARC-F may be a valuable indicator of mortality [56] and a predictor of the risk of sarcopenia or probable sarcopenia [175]. However, they caution against overinterpreting these findings, as the relationship between depression and malnutrition remains complex and not fully understood [176]. 

The multivariate model revealed that individuals with probable sarcopenia, as identified by the SARC-F tool, had a significantly higher risk of malnutrition (OR = 5.95, 95% CI: 1.29–31.47, *p* = 0.026). Additionally, a separate study developed a four-parameter model incorporating age, depression, disability, and physical fitness, which demonstrated high discriminatory accuracy in predicting malnutrition risk (AUC = 0.747; 95% CI: 0.686–0.808) [93].

The aforementioned studies highlight the impact of various physiological and pathological factors on the likelihood of malnutrition in older individuals. Consequently, malnutrition is a multifaceted issue that demands a comprehensive approach. In the context of the present study, we evaluated 44 potential determinants of malnutrition risk and identified nine key predictors that, when combined in a single model, provided the most accurate prediction of malnutrition risk in older adults.

The results demonstrated that the model is well suited for practical applications, offering consistent and accurate predictions. In practice, this model may serve as a valuable tool for data analysis, facilitating more effective assessment and management of malnutrition risk in older individuals.

### Limitations and Strengths of the Study

This study presents several strengths. Firstly, the use of a panel of nine parameters significantly improved the precision of malnutrition risk assessment. Secondly, the study underscores the importance of employing comprehensive methods to evaluate the health of older individuals, especially concerning malnutrition risk. These findings may stimulate further research aimed at identifying and validating additional parameters to enhance the accuracy of malnutrition risk assessments. However, additional research is necessary to explore the interdependence of different parameters and to refine the diagnostic panel for optimal performance.

The present study employs a comprehensive geriatric assessment that adopts a multidimensional approach to evaluate patients. This assessment encompasses a wide range of factors, including cognitive function, psychosocial aspects, physical health, basic vital functions, and daily living capabilities. Additionally, it includes evaluations of polypharmacy, sarcopenia, and sarcopenic obesity, providing a thorough overview of the patient’s health status.

It is important to acknowledge certain limitations of the study. Although patients were randomly selected from among willing participants, the study was confined to a group of seniors living in the community. As such, the findings may not be applicable to the broader older population. Additionally, the study represents a cross-sectional analysis at a specific point in time, which may not capture long-term changes in nutritional status. Consequently, the results may be particular to the study population and may not be generalizable to other age groups or demographic groups.

The cross-sectional design of the survey may limit the ability to draw conclusions about causal relationships, as the survey does not provide information about changes over time, the causality of phenomena occurring, or the chronology of the occurrence of events.

Although validated diagnostic tools were used, it is important to note that the GDS, CNAQ, SNAQ, and IADL were assessed using self-reporting questionnaires. As such, the results may be subject to measurement error due to the subjective nature of self-reporting. Furthermore, our study did not incorporate additional diagnostic tools specifically designed for assessing malnutrition risk in older individuals, which could have provided a more comprehensive evaluation. 

In future studies, incorporating biochemical biomarkers could enhance the assessment of nutritional status in elderly patients. Additionally, conducting long-term research with a larger and more diverse sample size would be beneficial. Such studies should account for external factors including social support, access to healthcare, economic status, physical activity, and dietary intake. This approach would enable the tracking of changes in nutritional status over time, providing a more comprehensive understanding of the dynamics involved. 

Conducting studies on the efficacy of nutritional, social, and psychological interventions tailored to the nine-factor model would be advantageous. These studies could provide valuable insights into designing more effective support programs for enhancing the nutritional status of older adults.

Furthermore, the findings in our study have significant practical implications. They can be utilized by nutritionists, physicians, diagnosticians, and other professionals involved in the care of older individuals to conduct comprehensive assessments of their nutritional status. The study offers tools and methods that assist in identifying and monitoring malnutrition risk, as well as developing tailored intervention strategies to meet the individual needs of patients.

## 5. Conclusions

The study demonstrates that while individual biomarkers such as phase angle (PA) and the geriatric depression scale (GDS) are effective in assessing malnutrition risk in older adults, a multi-parameter model could be considered a form of composite biomarker, providing a more integrated and accurate assessment of malnutrition risk by combining multiple relevant indicators. Validating and refining this expanded model across diverse populations and clinical settings will be essential for optimizing its practical application. Future research should build upon these findings by incorporating additional measures of malnutrition, including biochemical markers and other pertinent indicators of nutritional status.

## Figures and Tables

**Table 1:** The demographic characteristics of all participants and segmented by malnutrition risk according to MNA.

Trait	Total Sample, N = 154	Risk of Malnutrition		*p*
		Yes, N = 58	No, N = 96	
Sex				
Female N (%)	119 (77.27%)	48 (82.76%)	71 (73.96%)	0.207
Male N (%)	35 (22.73%)	10 (17.24%)	25 (26.04%)	
Age, years, median (Q1, Q3)	69.00 (65.00, 72.00)	69.00 (65.00,73.0)	69.00 (65.75,72.0)	0.74
Marital status				
Divorced, N (%)	27 (17.53%)	12 (20.69%)	15 (15.63%)	0.511
Married, N (%)	71 (46.10%)	24 (41.38%)	47 (48.96%)	
Single, N (%)	12 (7.79%)	3 (5.17%)	9 (9.38%)	
Widowed, N (%)	44 (28.57%)	19 (32.76%)	25 (26.04%)	
Education				
Higher, N (%)	73 (47.40%)	25 (43.10%)	48 (50%)	0.418
Primary, N (%)	9 (5.84%)	5 (8.62%)	4 (4.17%)	
Secondary, N (%)	72 (46.75%)	28 (48.28%)	44 (45.83%)	
Lifestyle				
Smoking status, N (%)	14 (9.09%)	8 (13.79%)	6 (6.25%)	0.115
Sleep duration, N (%)				
up to 6 h/day	42 (27.27%)	17 (29.31%)	25 (26.04%)	0.659
over 6 h/day	112 (72.73%)	41 (70.69%)	71 (73.96%)	
Diseases *				
Hypertension, N (%)	83 (53.90%)	30 (51.72%)	53 (55.21%)	0.674
Diabetes type 2, N (%)	22 (14.29%)	10 (17.24%)	12 (12.50%)	0.415
Liver disease, N (%)	14 (9.09%)	6 (10.34%)	8 (8.33%)	0.674
Heart disease, N (%)	40 (25.97%)	19 (32.76%)	21 (21.88%)	0.136
Hypothyroidism, N (%)	26 (16.88%)	13 (22.41%)	13 (13.54%)	0.154
Gout, N (%)	15 (9.74%)	8 (13.79%)	7 (7.29%)	0.187
Depression, N (%)	14 (9.09%)	9 (15.52%)	5 (5.21%)	0.031
Osteoporosis, N (%)	16 (10.39%)	10 (17.24%)	6 (6.25%)	0.030
Hyperlipidemia, N (%)	55 (35.71%)	22 (37.93%)	33 (34.38%)	0.655
COVID-19 in the past, N (%)	70 (45.45%)	23 (39.66%)	47 (48.96%)	0.261
Medication *				
Statins, N (%)	62 (40.26%)	27 (46.55%)	35 (36.46%)	0.216
Hypertensive medication, N (%)	82 (53.25%)	29 (50.00%)	53 (55.21%)	0.530
Oral diabetes medications, N (%)	21 (13.64%)	10 (17.24%)	11 (11.46%)	0.311
COVID-19 vaccine, N (%)	119 (77.27%)	48 (82.76%)	71 (73.96%)	0.207
SSRI/MAOI **, N (%)	17 (11.04%)	11 (18.97%)	6 (6.25%)	0.015
Hypothyroidism medication, N (%)	20 (12.99%)	8 (13.79%)	12 (12.50%)	0.817
Polypharmacy, N (%)	50 (32.5%)	24 (41.38%)	26 (27.08%)	0.066

* Hypertension is defined as a systolic blood pressure (SBP) of 140 mmHg or a diastolic blood pressure (DBP) of 90 mmHg, or the use of antihypertensive medication or a history of hypertension. Diabetes mellitus type 2 is defined as the use of oral antidiabetic drugs or insulin, or a history of diabetes mellitus. Liver disease is defined as the use of medication for liver disease or a medical diagnosis of liver disease, as documented in the patient’s history. Cardiovascular disease is defined as the use of cardiovascular medication or medical diagnosis declared in history. Hypothyroidism is defined as the use of levothyroxine or a medical diagnosis as stated in the patient’s history. Gout is defined as the use of uric acid-lowering drugs or a medical diagnosis declared by history. Depression is defined as the use of antidepressants or a medical diagnosis of depression. Osteoporosis is defined as the treatment for osteoporosis or medical diagnosis declared in history. Hyperlipidemia is defined as the the participant was taking medication to lower cholesterol levels. COVID-19 history is defined as the history of the detection of positive SARS-CoV-2 antibodies or a history of a positive SARS-CoV-2 test result. ** SSRI—selective serotonin reuptake inhibitors; MAOI—monoamine oxidase inhibitors.

**Table 2:** Physical fitness and body composition for the overall sample and by risk of malnutrition.

Trait	Total Sample, N = 154	Risk of Malnutrition		*p*
		Yes, N = 58	No, N = 96	
	Median (Q1, Q3)	Median (Q1, Q3)	Median (Q1, Q3)	
GS (kg)	20.10 (15.43, 25.69)	20.28 (15.40, 25.00)	20.05 (15.69, 27.88)	0.487
AO, N (%)	72 (46.75%)	27 (46.55%)	45 (46.88%)	0.969
BMI				0.747
Normal, N (%)	85 (55.19%)	34 (58.62%)	51 (53.13%)	
Obesity, N (%)	21 (13.64%)	8 (13.79%)	13 (13.54%)	
Overweight, N (%)	48 (31.17)	16 (27.59%)	32 (33.33%)	
WHR	0.84 (0.80, 0.91)	0.85 (0.81, 0.90)	0.84 (0.80, 0.94)	0.865
FDT				0.220
Android, N (%)	94 (61.04%)	39 (67.24%)	55 (57.29%)	
Gynoid, N (%)	60 (38.96%)	19 (32.76%)	41 (42.71%)	
MAC (cm)	29.00 (27.00, 31.75)	28.50 (26.00, 31.00)	29.00 (27.75, 32.00)	0.161
CC (cm)	36.00 (35.00, 38.00)	36.00 (34.25, 38.75)	37.00 (35.00, 38.00)	0.527
RFM (%)	40.17 (34.79, 45.28)	39.77 (34.89, 44.38)	40.65 (34.31, 45.39)	0.824
FFM (kg)	43.14 (38.89, 50.85)	41.01 (37.45, 47.68)	44.72 (40.03, 51.81)	0.037
SMM (kg)	20.15 (17.29, 24.43)	18.35 (16.79, 23.03)	20.98 (17.84, 24.91)	0.014
PMMTW (%)	27.24 (25.07, 30.39)	26.74 (25.40, 29.01)	27.46 (24.97, 31.13)	0.354
MM				0.053
High, N (%)	146 (94.81%)	52 (89.66%)	94 (97.92%)	
Low, N (%)	8 (5.19%)	6 (10.34%)	2 (2.08%)	
ASMI (kg/m^2^)	7.67 (6.86, 8.98)	7.27 (6.62, 8.63)	7.95 (7.14, 9.18)	0.020
TBW (L)	32.67 (29.26, 38.01)	31.11 (28.09, 35.78)	34.00 (30.04, 38.78)	0.041
ECW (L)	15.44 (13.87, 17.21)	15.03 (13.62, 16.53)	15.75 (14.06, 17.29)	0.141
FFMI (kg/m^2^)	16.81 (15.45, 18.99)	16.43 (14.96, 18.48)	17.17 (15.66, 19.14)	0.051
FMI (kg/m^2^)	10.99 (8.70, 13.47)	10.87 (8.38, 13.32)	11.14 (8.75, 13.53)	0.514
PA (degrees)	5.31 (5.02, 5.82)	5.15 (4.95, 5.57)	5.49 (5.07, 5.97)	0.008
VAT (L)	1.84 (1.31, 2.47)	1.79 (1.31, 2.40)	1.87 (1.25, 2.56)	0.811

Note: GS—grip strength: average of two measurements; right and left hand (kg). AO—abdominal obesity. BMI—body mass index. WHR—waist–hip ratio. FDT—fat distribution type. MAC—mid-arm circumference. CC—calf circumference. RFM—relative fat mass. FFM—fat-free mass. SMM—skeletal muscle mass. PMMTW—proportion of muscle mass in total weight. MM—muscle mass. ASMI—appendicular skeletal muscle mass index level. TBW—total body water. ECW—extracellular water. FFMI—fat-free mass index. FMI—fat mass index. PA—phase angle. VAT—visceral adipose tissue.

**Table 3:** Energy and metabolism and nutritional and functional assessment.

Trait	Total Sample N = 154	Risk of Malnutrition		*p*
		Yes, *n* = 58	No, *n* = 96	
	Median (Q1, Q3)	Median (Q1, Q3)	Median (Q1, Q3)	
TEE (kcal/day)	2197.17 (2028.97, 2410.06)	2173.83 (1996.63, 2308.88)	2217.52 (2043.36, 2443.14)	0.114
REE (kcal/day)	1348.71 (1255.39, 1480.11)	1297.85 (1241.09, 1442.25)	1360.97 (1283.55, 1487.73)	0.065
TEC (kcal)	317,415.55 (270,696.0, 387,667.5)	310,418.40 (257,826.68, 371,170.7)	332,081.30 (279,880.1, 389,083.4)	0.259
GDS score [0–30]	4.00 (1.00, 9.00)	7.50 (2.00, 11.00)	3.00 (1.00, 5.25)	<0.001
TUG (s)	9.89 (8.92, 11.68)	10.59 (9.16, 12.29)	9.51 (8.78, 11.28)	0.015
SNAQ low score, N, (%)	46 (29.87%)	26 (44.83%)	20 (20.83%)	0.002
CNAQ low score, N, (%)	67 (43.51%)	38 (65.52%)	29 (30.21%)	<0.001
SARC F low score, N, (%)	15 (9.74%)	11 (18.97%)	4 (4.17%)	0.003

Note: TEE—total energy expenditure. REE—resting energy expenditure. TEC—total energy content. GDS—geriatric depression scale. TUG—timed up-and-go test. SNAQ—simplified nutritional appetite questionnaire. CNAQ—council on nutrition appetite questionnaire. SARC-F—strength, assistance in walking, rise from a chair, climb stairs, and falls questionnaire.

**Table 4:** Optimal cut-off points for numerical parameters and corresponding metrics for classifying the occurrence of malnutrition risk.

Parameter	Cut-Off Point	Accuracy	Sensitivity	Specificity	AUC
GS	≥20.15 kg	0.53	0.53	0.53	0.47
WHR	≥0.81	0.49	0.78	0.31	0.49
MAC	≤26.00 cm	0.63	0.28	0.84	0.57
CC	≤33.00 cm	0.62	0.22	0.85	0.53
BMI	≤25.83 kg/m^2^	0.66	0.41	0.80	0.56
RFM	≤41.61%	0.52	0.67	0.43	0.51
FFM	≤42.83 kg	0.62	0.64	0.60	0.60
SMM	≤19.33 kg	0.62	0.62	0.63	0.62
PMTW	≤29.05%	0.53	0.78	0.39	0.54
ASMI	≤6.91 kg/m^2^	0.66	0.41	0.81	0.61
TBW	≤32.28 L	0.62	0.62	0.61	0.60
ECW	≤15.13 L	0.59	0.63	0.63	0.57
FFMI	≤15.45 L kg/m^2^	0.64	0.36	0.81	0.59
FMI	≤11.62 L kg/m^2^	0.53	0.66	0.46	0.53
PA	≤5.72°	0.58	0.86	0.42	0.62
VAT	≤2.05 L	0.53	0.69	0.43	0.51
TEE	≤2336.13 kcal	0.54	0.78	0.40	0.58
REE	≤1297.85 kcal	0.64	0.52	0.71	0.59
TEC	≤285,067 kcal	0.60	0.40	0.73	0.55
GDS [0–30]	≥5.00	0.67	0.67	0.68	0.69
TUG	≥9.61 s	0.58	0.67	0.63	0.62

Note: AUC—area under the curve. GS—grip strength. WHR—waist–hip ratio. MAC—mid-arm circumference. CC—calf circumference. BMI—body mass index. RFM—relative fat mass. FFM—fat-free mass. SMM—skeletal muscle mass. PMTW—proportion of muscle mass in total weight. ASMI—appendicular skeletal muscle mass index level. TBW—total body water. ECW—extracellular water. FFMI—fat-free mass index. FMI—fat mass index. PA—phase angle. VAT—visceral adipose tissue. TEE—total energy expenditure. REE—resting energy expenditure. TEC—total energy content. GDS—geriatric depression scale. TUG—timed up-and-go.

**Table 5:** Regression coefficients of the fitted generalized linear model after applying the backward stepwise algorithm, *N_obs_* = 154, *R*^2^*_Tjur_* = 0.344.

Predictors	The Occurrence of the Risk of Malnutrution		
	OR	CI 95%	*p*
(Intercept)	0.14	0.07–0.28	<0.001
Smoking [no]	Reference category		
Smoking [yes]	7.08	1.90–28.65	0.004
Osteoporosis [no]	Reference category		
Osteoporosis [yes]	4.89	1.34–19.49	0.019
SSRI/MAOI medication [no]	Reference category		
SSRI/MAOI medication [yes]	4.42	1.11–19.64	0.040
Grip strength average of two measurements; right and left hand (centered by the optimal cutoff value = 20.15 kg)	1.07	1.00–1.15	0.068
Skeletal muscle mass (centered by the optimal cutoff value = 19.33 kg)	0.86	0.76–0.95	0.008
GDS score (centered by the optimal cutoff value = 5.0)	1.07	0.99–1.16	0.077
Timed up-and-go test result (centered by the optimal cutoff value = 9.61 s)	1.22	1.00–1.50	0.052
CNAQ normal appetite	Reference level		
CNAQ decreased appetite	4.34	1.89–10.46	0.001
SARC F no risk of sarcopenia	Reference level		
SARC at risk of sarcopenia	5.95	1.29–31.47	0.026

## Data Availability

Data are contained within the article.

## Appendix A. Specification of the Parameters Used in the Regression Model

**Table A1:** Parameters incorporated and excluded in the initial full regression model prior to optimization using the backward step algorithm.

Group of Parameters	Parameters	
	Incorporated in Regression Model	Not Used Due to Multicollinearity
Demographic	sex	marital status
	age	
	BMI (categories)	
	education	
Lifestyle and behavioral factors, comorbidities, or historical illnesses	smoking	COVID-19 in the past
	daily sleep duration	
	HT	
	DM	
	liver disease	
	heart disease	
	hypothyroidism	
	gout	
	depression	
	osteoporosis	
	hyperlipidemia	
Ongoing medication and treatments	statins	-
	hypertension medications	
	oral diabetes medications	
	COVID-19 vaccine	
	SSRI/IMAO	
	hypothyroidism drugs uthyrox/letrox	
	Osteoporosis drugs	
	polypragmasy (taking more than five drugs)	
Physical fitness and body composition	grip strength average of two measurements; right and left hand (kg)	extracellular water value (L)
	abdominal obesity	FMI index (kg/m^2^)
	WHR	relative fat mass value (%)
	fat distribution type	ASMI (kg/m^2^)
	MAC right arm circumference (cm)	
	right calf circumference (cm)	
	fat-free mass value (kg)	
	skeletal muscle mass (kg)	
	proportion of muscle mass in total weight (%)	
	muscle mass	
	total body water value (L)	
	FFMI (kg/m^2^)	
	phase angle (degrees)	
	visceral adipose tissue (L)	
Energy, metabolism, and nutritional assessment	total energy expenditure (kcal)	resting energy expenditure (kcal)
	energy content (kcal)	
	GDS score [0–30]	
	time for up-and-go (s)	
	SNAQ risk	
	CNAQ risk	
	SARC F	

## Appendix B. The Multicollinearity Analysis

**Table A2:** The results of the multicollinearity for the regression model predictors.

Predictor	VIF	
	Value	CI 95%
smoking	1.13	1.03–1.53
osteoporosis	1.09	1.02–1.59
SSRI/IMAO medicine intake	1.07	1.01–1.70
grip strength average of two measurements right and left hand	2.28	1.86–2.92
skeletal muscle mass	2.43	1.97–3.11
GDS score	1.11	1.02–1.55
timed up-and-go test result	1.27	1.11–1.61
CNAQ risk	1.12	1.03–1.53
SARC F	1.09	1.01–1.62

Note: CI 95%—confidence interval 95%.

## Appendix C. Validation Results of the Model Fit to Observed Data

**Table A3:** Validation results of model fit to observed data.

Test	Statistic	Model Logit		
		Value	*df*	*p*
HL	χ^2^	3.08	6	0.799
mHL	F	1.09	7	0.373
OsRo	Z	1.74	-	0.083
SstPgeq0.5	Z	0.19	-	0.850
SstPl0.5	Z	1.09	-	0.275
SstBoth	χ^2^	1.22	2	0.542
SllPgeq0.5	χ^2^	0.04	1	0.843
SllPl0.5	χ^2^	1.11	1	0.291
SllBoth	χ^2^	1.37	2	0.504

Note: HL—Hosmer–Lemeshow test; mHL—modified Hosmer–Lemeshow test; OsRo—Osius and Rojek’s test; SstPgeq0.5—standardized score test for predictions greater than or equal to 0.5; SstPl0.5—standardized score test for predictions less than 0.5; test SstBoth—sum of standardized score test for binary outcomes; test SllPgeq0.5—sum of logged likelihood for predictions greater than or equal to 0.5; test SllPl0.5—sum of logged likelihood for predictions less than 0.5; test SllBoth—combination of SllPgeq0.5 and SllPl0.5; *df*—degrees of freedom; *p*—the *p*-value of statistical test. “-” means “not applicable”.
